# Sustainable Development Goals and the Mental Health of Resettled Refugee Women: A Role for International Organizations

**DOI:** 10.3389/fpsyt.2019.00608

**Published:** 2019-08-30

**Authors:** Helen Herrman

**Affiliations:** Orygen, The National Centre of Excellence in Youth Mental Health, Centre for Youth Mental Health, The University of Melbourne, Parkville, VIC, Australia

**Keywords:** refugee women, mental health, sustainable development goals, empowerment, mental health promotion

## Introduction

Refugee women and girls typically face enormous adversities whether resettled in high-income or low-income countries. Women and men alike flee from their homes because they have endured terrible events in their countries of origin. They often face more experiences of violence, imprisonment and/or deprivation during their flight and in places of temporary shelter. These experiences can be expected to have adverse effects on their mental health, including a high risk of mental ill health. When finally resettled, all too often their ordeal is not finished as they face the demands of life in a new place, whether alone or with family responsibilities (1).

The conditions and experiences that refugee women encounter on resettlement have a significant influence on their mental health, either supporting its maintenance and recovery or else compounding the adverse effects of previous experiences (1). The government policies and the resources and services available to support the settlement of refugees, and above all, community attitudes are crucial to the health of these women and the subject of intense debate in countries around the world. Strategies that are likely to (1) promote the mental health of refugee women and girls and (2) tackle their mental health problems overlap with each other. They cover investment in health and social services, including workforce development and research, as well as the broader alignment of social policies and practices across education, employment, family welfare, and community planning to support good mental health outcomes. Key to the success of these strategies as in any community development work is the empowerment and participation of the resettled women, and the development of partnerships in local communities.

The Sustainable Development Goals (SDGs) endorsed by all United Nations member states in 2015 can be used to link these strategies to the broader goals of sustainable human development in countries. There are good indications of synergy between the SDGs and many of the key social determinants of mental disorders. The goals are also relevant to the treatment and care of the women with mental ill health (3). The SDGs provide a framework for national and international interventions relevant to mental health and mental disorders among refugee women, whether the women are resettled in high-income or low-income countries.

### The Role of International Organizations

International organizations, have a major role in raising awareness of the adverse effects on mental health of life as a refugee. These organizations, whether inter-governmental, non-governmental or philanthropic, can set expectations and inform policies and plans relevant to resettled refugee women in countries and communities [e.g., Ref. (2)] and especially draw attention to the links with and mutual benefits of meeting the SDGs. This is the primary focus for this article. International organizations are also responsible for drawing attention to the mental health consequences of the factors that drive people from their homes, including poverty and insecurity during and after conflict and emergencies, and the effects of climate change on generating these.

### Poverty, Violence, and Discrimination Against Refugee Women and Girls

All women with mental ill health face a double disadvantage, from gender-based stigma and discrimination, and from the stigma and discrimination related to mental illnesses (4). This is compounded for refugee women by the associated adversities, especially when they experience violence and gender-based discrimination. They frequently face poor access to education, health care, employment, and other assistance as they are resettled. The violence increases the risk of depression, anxiety, trauma symptoms, suicidal ideas, and substance abuse (5). Human rights abuses during conflict, emergencies and displacement, intimate partner violence, and insecurity and poverty are all likely contributors to the high risk of perinatal depression and other mental health problems for refugee women.

*“The main reason for what is now nothing less than an epidemic of assault on women, which occurs at every age and stage of life, is the imbalance of power between men and women—discriminatory laws and policies, inequitable practices, stigma, shame, silence, submissive femininity, and dominant masculinity”* (6).

Yet mental health among refugee women is still seen in many places as a private matter rather than a challenge for social and economic policy. Effective public health responses remain inadequate in many settings. Commenting on the global challenge of women’s health and women’s rights, Stephen Leeder (7) notes the basic problem of *“… a pathological view of women—that they are not a priority and that public resources should be invested elsewhere.”*

### The Mental Health of Refugee Women and Girls and the Sustainable Development Goals (SDGs)

Mental health is directly acknowledged in SDG3, which emphasizes the inclusion of mental health care in universal health coverage. With the human suffering and financial costs associated with mental disorders among refugee women, investments in the treatment and care of these women have the potential to increase significantly their capabilities and productivity (3). However, the burden of these disorders among refugee women is unlikely to be relieved by improved access to mental health treatment alone. There is growing global evidence that adverse social and economic circumstances, including poverty, gender discrimination, violence, and forced migration have a strong influence on the risk of mental disorders. Actions in various spheres of community life such as education, employment, and family welfare, that are also needed to meet the relevant SDGs (including SDG5 related to gender discrimination), have the potential to reduce the burden of mental disorders among refugee women by addressing their upstream social determinants (3). Specific policies and plans concerning resettlement arrangements and targeted services and welfare provisions are also important for the mental health of refugee women and hence relevant to country plans to meet several of the SDGs (1, 2).

### Promoting the Mental Health of Refugee Women and Girls

Partnerships within and between health and non-health sectors are needed to support the human rights of the women and reduce exposure to adversity including violence, discrimination, and poor access to education and income-generating work. UN Women emphasizes women’s empowerment in its policies and programs, and Melinda French Gates writes that *“… empowered women have the potential to transform their societies …. helping women and girls realize their own power to advance the wellbeing of their families, their communities, and their societies”* (8).

The wellbeing of refugee women and their empowerment and participation are important contributors to a safe and constructive community life. Their participation is critical for tackling the social and health concerns of their peers—such as maternal and child health, violence at home and in the streets, substance abuse, and gender equity. Participation and the empowerment underlying it are in turn components of good mental health (4, 9).

Gender and cultural sensitivity are needed in service planning and the training of health and social service workers. In many communities, the services provided for refugee women and girls with mental ill health in primary health care, maternal and child health services, community mental health services, or hospital settings do not respond adequately to their needs. This is best achieved when mental health professionals, health workers, and researchers join the women and community partners to advocate for these strategies and implement these approaches (5).

## Discussion

International organizations are in a position to support the advocacy and training for this work. They can also bring to attention the effects of specific post-migration factors on mental health. The role of the government of a country to respect, uphold, and administer the 1951 United Nations Refugee Convention with fairness and promptness; to act with humanity and compassion; and to protect individual cultural, religious, and spiritual dignity provides a basis for positive community attitudes toward support and care for refugees (2). Government policies on family separation and reunification and the granting of citizenship rights are highly relevant to the experience of resettlement, along with the targeted provision of services of various types. Resettlement services provided in a numtber of receiving countries include specialist services for survivors of torture and trauma, health and settlement services for newly arrived refugees, language and cultural support in educational settings, and support for refugee families experiencing conflict and tension (1). Services such as these are likely to have significant and lasting benefits for mental health as well as support the SDGs.

Local contextual solutions are the best way to support the mental health of refugee women. They can be developed through assessing needs and designing strategies along with participants in each country and community involved, and monitoring and evaluating the implementation of changes and projects. There is an urgent need for adequately powered longitudinal studies on the mental health of refugee women that integrate social, economic, and biological data, especially in low-income and middle-income settings, and studies of implementing social and health interventions in these settings (3).

## Conclusions

Improving the mental health of refugee women and girls is a significant topic of interest for international organizations including the World Health Organization, the International Organization for Migration, UN Women and other UN agencies, and civil society organizations such as the World Psychiatric Association. The SDGs provide a framework for advocacy and advice at international and national levels. This encourages cross-sectoral action with participation of the women to promote mental health, prevent and treat mental illnesses, and support research on these interventions.

## Author Contributions

HH drafted the original opinion.

## Conflict of Interest Statement

The author declares that the research was conducted in the absence of any commercial or financial relationships that could be construed as a potential conflict of interest.
